# Association Between Ideal Cardiovascular Health and Executive Function in Chinese Primary School Children

**DOI:** 10.3389/fpubh.2021.736424

**Published:** 2022-01-12

**Authors:** Zhaohuan Gui, Li Cai, Yajie Lv, Lijuan Lai, Xia Zeng, Yajun Chen

**Affiliations:** Department of Maternal and Child Health, School of Public Health, Sun Yat-sen University, Guangzhou, China

**Keywords:** child, cognition, epidemiology, lifestyle, risk factors

## Abstract

**Aims:** Little information exists on the associations of cardiovascular health, a new metric proposed by the American Heart Association, and executive function, particularly in children. We aimed to explore this topic.

**Methods:** We studied 3,798 children aged 6–12 years from 5 schools in Guangzhou, China. The executive function of children was evaluated using parent reports of the Behavioral Rating Inventory of Executive Function, which included 2 composite indexes and 8 subscale scores. We calculated the number of ideal cardiovascular health (range: 0–7) based on smoking, body mass index, physical activity (PA), diet, blood pressure, cholesterol, and glucose. A generalized linear mixed model was used to assess the association of the number of ideal cardiovascular health metrics and executive function.

**Results:** Compared with children exhibiting 1–3 ideal cardiovascular health metrics, decreases of 1.37–2.63 points (indicating better performance) in metacognition index and its 5 subscale indexes (initiate, working memory, plan/organize, organization of materials, and monitor) were observed in children who attained 5 or 6–7 ideal metrics (all *p* for trend <0.001). Ideal diet and ideal PA were independently associated with lower indexes of behavioral regulation and metacognition.

**Conclusions:** The number of ideal cardiovascular health was positively associated with performance of executive function in children.

## Introduction

A construct of ideal cardiovascular health, also referred as life's simple 7, is defined by the American Heart Association (AHA) in 2010 to track health status in relation to a 2020 strategic goal to improve cardiovascular health of Americans (1). Cardiovascular health is the simultaneous presence of 4 modifiable health behaviors [body mass index (BMI), smoking, physical activity (PA), and diet] and 3 modifiable health factors [blood pressure (BP), total cholesterol (TC), and fasting blood glucose]. Previous evidence has indicated that the status of cardiovascular health during childhood is a strong predictor for cardiovascular health and future subclinical cardiovascular disease (CVD) development in adulthood (2). Over the past decades, the global prevalence of ideal cardiovascular health in youth was low (3), with more severe situation in developing countries (4).

Additionally, cardiovascular health plays a critical role in brain health (5). Executive function refers to a top-down mental process that encompasses self-control, working memory, cognitive flexibility, reasoning, and problem solving (6). These high-order skills of executive function develop dramatically across childhood and were related to learning and academic achievement as well as life quality in adulthood (7, 8). Many epidemiological studies have explored the relationship between poor diet, physical inactivity, obesity, and hypertension, individually or in various combination, with deficits in the domain of executive function in children, albeit the inconsistent results exist (9–11). Moreover, they mainly focused on either fewer metrics or individual risk factor, evidence concerning the association between the AHA-defined clusters of cardiovascular health metrics with executive function is still sparse. Since cardiometabolic risk factors tend to occur together more frequently than expected by chance alone and may share similar physiological pathways (12), an overall ideal cardiovascular health profile may be more effective in improving the executive performance than any single factor. Several previous studies have investigated the relationship of the AHA's cardiovascular health metrics and cognitive function (13–21). These studies were conducted in adults or elderly population and reported beneficial effects of increasing number of ideal health metrics on cognitive performance (13–21). However, no literature on this topic in children has been performed who are more susceptible because their brain has profound plasticity.

Therefore, the objective of this study was to investigate the associations between the AHA construct of ideal cardiovascular health metrics and executive function in the Chinese children. We hypothesized that a greater number of cardiovascular health metrics would be associated with executive performance. Furthermore, each metric included in the cardiovascular health would be related to executive function.

## Methods

### Population and Study Design

This analysis was embedded on the baseline data from an ongoing population-based prospective cohort study in Guangzhou, China (Clinical Trial Registration: June 27, 2018; Registration number: NCT03582709), previously described in detail (22). Briefly, in 2017, a multistage random sampling method was used to recruit participants. First, five districts (3 in urban area and 2 in suburb) in Guangzhou city were randomly selected. Second, one elementary school within each study district was randomly selected, and a total of 5 elementary schools were included. Third, all students of the 5 schools were invited to participate in this study.

The above sampling strategy generated 8,324 eligible participants, of whom 5,788 children aged 6–12 years received anthropometric measurements and questionnaire survey. A total of 4,744 children provided voluntarily a blood sample. We additionally excluded 946 students without information on the Behavioral Rating Inventory of Executive Function (BRIEF) or important covariates (i.e., demographic information and lifestyles). Finally, 3,798 participants were included in the data analysis. All participants and their parents/guardians gave their written informed consent before participation, and this study was approved by the Ethical Review Committee for Biomedical Research, Sun Yat-sen University, Guangzhou, China.

### Ascertainment of Health Behaviors and Factors

We measured cardiovascular health metrics based on the definitions published by the AHA (1). Due to lack of information in the questionnaire, we modified the definition for diet component in the study (Supplementary Table S1). The following 7 metrics were classified into ideal vs. not ideal. Body height and weight were measured in accordance with standardized procedures with participants lightly dressed and in bare feet with a fixed stadiometer (model TZG, Hongya science & education, Jiangyin, China) and a calibrated weighing scale (model Seca-899, Seca Medical Measuring Systems and Scales, Hangzhou, China) (22). BMI was calculated as weight in kilograms divided by height in meters squared. Ideal BMI was defined as <85th percentile based on the Working Group of Obesity in China sex specific BMI-for-age growth charts for youths aged 7–18 years (23). Information on smoking status was collected by a question “How many times did you smoke in the past 7 days?” and ideal smoking status was defined as never smoking. Children reported the frequency and the duration of vigorous-intensity PA (i.e., running, basketball, football, and swimming) and moderate-intensity PA (i.e., cycling, table tennis, badminton, and calisthenics) over the preceding 7 days for a minimum of 10 min. Moderate-to-vigorous-intensity PA (MVPA) was calculated by summing up the daily time of the two kinds of PA. Children who on average engaged in ≥60 min of MVPA per day were classified as having an ideal PA level (24). Dietary intake for the past 7 days was assessed *via* frequency food questionnaire, and we replaced grain and salt, two components proposed by the AHA, with milk, beans or dairy-or bean-products, and fried food consumption, respectively. The healthy diet score was defined to include the following 5 components: (1) fruits and vegetables (≥1 times/day); (2) fish or fish products (≥2 times/week); (3) milk, beans, or dairy-or bean-products (≥1 times/day), (4) fried food (≤ 2 times/week); and (5) sugar-sweetened beverages (SSB) (≤ 2 times/week). Ideal diet was defined as having 4 or 5 healthy components.

Blood samples were collected between 7 and 9 a.m. at physical examination with children being asked to fast for at least 10 h before the test. Additionally, parents or guardians were informed by head teachers to make sure their children to fast completely for 10 h. A venous blood sample (5 ml) was taken from the antecubital vein. Samples were stored in sterile blood collection tubes in refrigerated conditions (4–8°C) for not more than 4 h after collection and then sent to an analytical laboratory for testing. Fasting blood glucose concentrations were measured by the glucose oxidase method (HITACHI 7180 automated biochemistry analyzer, Tokyo, Japan), and TC were analyzed by enzymatic assays (HITACHI 7180 automated biochemistry analyzer, Tokyo, Japan). Ideal fasting blood glucose and TC concentrations were classified as <5.6 and <4.4 mmol/L, respectively.

After 10 min rest, systolic BP (SBP) and diastolic BP (DBP) were measured with a validated electronic sphygmomanometer (OMRON HEM-7071, Japan) with children seated. The average of 2 readings was used. Ideal BP was defined as SBP and DBP <90th percentile according to the age-, sex-, and height-specific standards for the Chinese children and adolescents (25).

The number of simultaneous presence of ideal cardiovascular health components for each participant was summed, ranging from 0 (worst cardiovascular health) to 7 (best cardiovascular health).

### Executive Function Evaluation

The execution function of children was evaluated using the parental form of BRIEF, which is designed to provide an assessment of behavioral executive difficulties in everyday life at 5 and 18 years of age (26). The BRIEF is used extensively in epidemiological studies and has been translated and validated for use in the Chinese children, with a high value of Cronbach's α from 0.70–0.96 (27). It consists of 86 items with a 3-point scale (1 for “never,” 2 for “sometimes,” and 3 for “very often”), which generate 2 broader indexes and 8 non-overlapping subscales. The behavior regulation index (BRI) incorporates 3 subscales: inhibit, shift, and emotional control. The metacognition index (MI) consists of 5 subscales: initiate, working memory, plan/organize, organization of materials, and monitor. Scores were age and sex-normed to a *t*-distribution [a mean of 50 and SD of 10]. A higher score is associated with more problems related to executive function.

### Covariates

A questionnaire was used to collect information on the demographic information and lifestyles of children, such as age (years), sex (boys or girls), only child (no/yes), parental education (highest level of either parent: below senior high school, senior high school, college, or university or above), monthly household income (<5,000 RMB/month, 5,000–7,999 RMB/month, 8,000–14,999 RMB/month, 15,000 or above, and not clear), and screen time. Screen time was calculated based on the total time of child's TV viewing, video game play, and computer use over the preceding 7 days. Screen time was dichotomized (<2 h/day or ≥2 h/day) in accordance with the guidelines of the American Academy of Pediatrics (28). We have drawn a directed acyclic graph (DAG) using DAGitty v3.0 software (www.dagitty.net) to identify the most parsimonious confounders set (29) (**Figure S1**). Finally, the following variables were selected as confounders in the main model: age, sex, only child, parental education, monthly household income, and screen time. Parents also completed the Strengths and Difficulties Questionnaire (SDQ) about child behavioral problems (used for stratified sensitivity analysis) (30). Recommended cut-off points for SDQ were used to identify children scoring in the “normal” and “borderline/abnormal” (30).

### Statistical Analysis

Due to the limited number of participants with lower or upper ideal heath scores, children scored 1–3 and 6–7 in ideal health metrics were combined to stabilize our estimates. Therefore, ideal cardiovascular health scores were divided into 4 categories: 1–3 points, 4 points, 5 points, and 6–7 points (no participants scored 0). Differences in basic characteristics according to the ideal health metrics classification were tested using one-way ANOVA and chi-square test for continuous and categorical variables, respectively.

We evaluated the association of total number of ideal health metrics and executive function using generalized linear mixed models (GLIMMIX). Model estimates were presented as point estimates with 95% *CI*s. Age, sex, only child, parental education, monthly family income, and screen time were adjusted as covariates in the model, and school was considered as a random effect. In the sensitivity analyses, we used the standard to screen for overweight and obesity among school-age children and adolescents in 2018 to repeat the results. In addition, we used GLIMMIX to evaluate the associations between each of 7 cardiovascular health metrics individually (categorized as ideal vs. not ideal) and 2 composite scores of BRIEF (BRI and MI). We further evaluated the potential effect modification of sex (boys or girls) and SDQ behavioral problems (normal, borderline/abnormal) through the addition of multiplicative interaction term between sex/behavioral problems and cardiovascular health scores into regression model.

All statistical analyses were performed using SAS version 9.4 (SAS Institute, Cary, NC, USA). A *p* < 0.05 was considered as statistically significant for a two-tailed test.

## Results

### Descriptive Statistics

**Figure 1:**
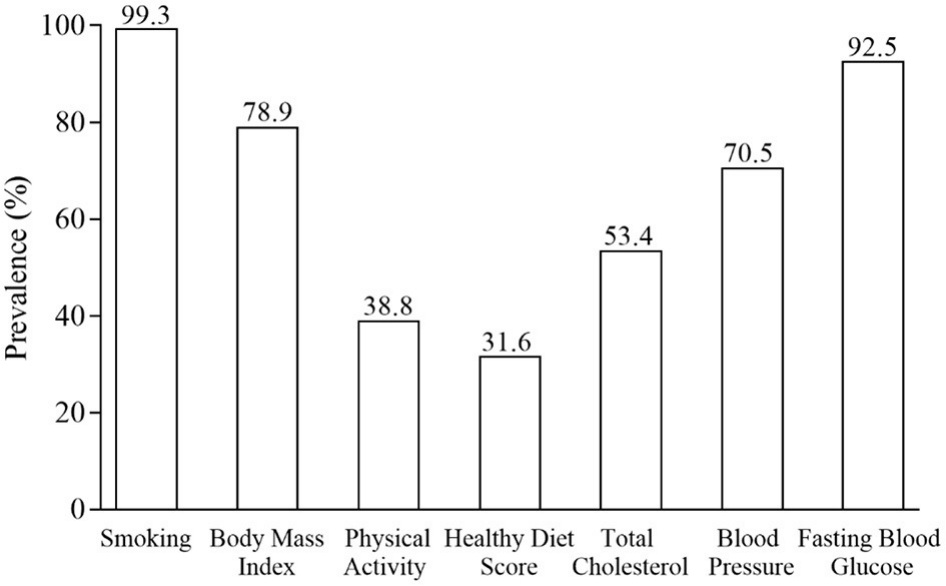
Prevalence of individual ideal cardiovascular health components (*n* = 3,798).

**Figure 2:**
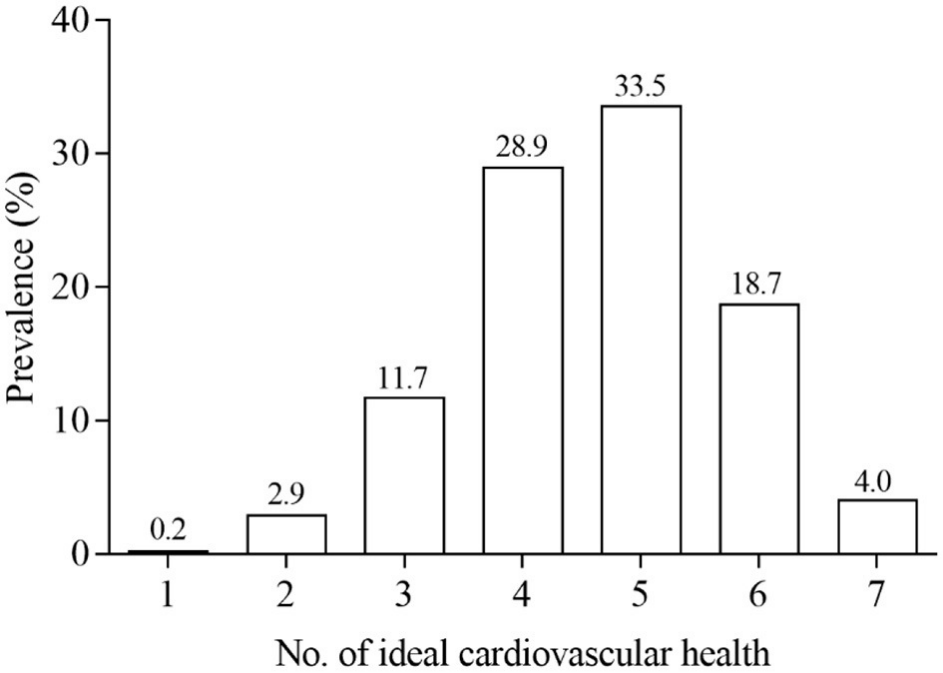
Prevalence of ideal cardiovascular health components (*n* = 3,798).

The characteristics of participants across the number of ideal cardiovascular health metrics are presented in Table 1. Participants were on average of 9.1 years old and 47.8% were girls. Older children and girls were more likely to meet greater number of ideal health components (*p* < 0.05). The prevalence of ideal health for the individual and total number of components are displayed in Figures 1, 2, Supplementary Tables S2, S3. Over half of study participants reported ideal smoking (99.3%), ideal fasting blood glucose (92.5%), ideal BMI (78.9%), ideal blood pressure (70.5%), and ideal total cholesterol (53.4%), while less than half of children exhibited ideal PA (38.8%) and ideal diet (31.6%) (Figure 1). Overall, none of children had 0 health metrics at ideal levels, and majority of children (62.4%) exhibited 4 or 5 ideal health components (Figure 2). The participants who met all 7 metrics of ideal health was only 4.0%. The scores distribution of BRIEF is shown in Supplementary Table S4. Included participants (*n* = 3,978) did not differ by age, only child, parental education, monthly household income, or screen time from the excluded participants (*n* = 946) (Supplementary Table S5). There was a high proportion of girls in the included children than the excluded.

**Table 1:** Study population characteristics by the number of ideal cardiovascular health metrics.

**Characteristics**	**Total (*n =* 3,798)**	**No. of ideal cardiovascular health metrics**			
		**1–3 points** **(*n =* 565, 14.9%)**	**4 points** **(*n =* 1,099, 28.9%)**	**5 points** **(*n =* 1,271, 33.5%)**	**6–7 points** **(*n =* 863, 22.7%)**
Age, year (mean ± SD)^a^	9.1 ± 1.7	9.0 ± 1.6	9.0 ± 1.7	9.1 ± 1.7	9.2 ± 1.8
Sex, *n* (%)^a^					
Boys	1,981 (52.2)	345 (17.4)	573 (28.9)	644 (32.5)	419 (21.2)
Girls	1,817 (47.8)	220 (12.1)	526 (29.0)	627 (34.5)	444 (24.4)
Only child, *n* (%)	
Yes	2,052 (54.4)	304 (14.8)	615 (30.0)	677 (33.0)	456 (22.2)
No	1,722 (45.6)	256 (14.9)	477 (27.7)	587 (34.1)	402 (23.3)
SDQ behavioral problems, *n* (%)					
Normal	3,205 (84.4)	474 (14.8)	922 (28.8)	1,068 (33.3)	741 (23.1)
Borderline/abnormal	329 (9.3)	48 (14.6)	100 (30.4)	123 (37.4)	58 (17.6)
Parental education, *n* (%)					
Below senior high school	131 (3.5)	22 (16.8)	32 (24.4)	50 (38.2)	27 (20.6)
Senior high school	522 (14.0)	91 (17.4)	148 (28.4)	161 (30.8)	122 (23.4)
College	854 (22.8)	125 (14.6)	243 (28.5)	288 (33.7)	198 (23.2)
University or above	2,232 (59.7)	316 (14.2)	657 (29.4)	751 (33.7)	508 (22.8)
Family income, RMB/month, *n* (%)					
4,999 or below	821 (22.0)	118 (14.4)	250 (30.5)	267 (32.5)	186 (22.7)
5,000–7,999	994 (26.6)	151 (15.2)	263 (26.5)	358 (36.0)	222 (22.3)
8,000–14,999	967 (25.9)	144 (14.9)	263 (27.2)	344 (35.6)	216 (22.3)
15,000 or above	490 (13.1)	62 (12.7)	147 (30.0)	160 (32.7)	121 (24.7)
Not clear	460 (12.3)	76 (16.5)	157 (34.1)	129 (28.0)	98 (21.3)
Screen time, *n* (%)					
<2 h	3,247 (86.8)	478 (16.3)	930 (30.7)	1,098 (31.1)	741 (22.0)
≥2 h/day	492 (13.2)	80 (14.7)	151 (28.6)	153 (33.8)	108 (22.8)

*SDQ, Strengths and Difficulties Questionnaire*.

a *Statistically significant difference among number of ideal cardiovascular health metrics (p < 0.05)*.

### Overall Cardiovascular Health Status and Executive Function

The associations between the number of ideal cardiovascular health metrics and executive function are summarized in Table 2. In crude model, children who exhibited 5–7 ideal health metrics had lower scores (indicating better performance) in 2 broader indexes (BRI and MI) and all 8 subscale indexes (inhibit, shift, emotional control, initiate, working memory, plan/organize, organization of materials, and monitor) than those who exhibited ≤ 3 ideal health metrics. However, the statistical significance of the associations remained only for MI and its 5 subscale indexes after adjustment for age, sex, only child, parental education, family income, and screen time. Compared with children exhibiting ≤ 3 ideal health metrics, decreases of 1.37–2.63 points in MI and its 5 subscale indexes (initiate, working memory, plan/organize, organization of materials, and monitor) were observed in children who attained 5 or 6–7 ideal health metrics (all *p* for trend <0.001).

**Table 2:** Association (95% *CI*) between the number of ideal cardiovascular health metrics and executive function (*n* = 3,798).

**Executive function**	***β*** **(95% confidence interval)**				
	**1–3 points**	**4 points**	**5 points**	**6–7 points**	***P* for trend**
**Crude model**					
BRI	0 (Ref.)	−0.08 (−1.11, 0.95)	−1.13 (−2.14, −0.13)^a^	−1.50 (−2.58, −0.42)^a^	0.0004
Inhibit	0 (Ref.)	−0.19 (−1.21, 0.83)	−1.08 (−2.08, −0.08)^a^	−1.38 (−2.45, −0.30)^a^	0.001
Shift	0 (Ref.)	−0.04 (−1.07, 0.98)	−0.82 (−1.82, 0.19)	−1.37 (−2.45, −0.29)^a^	0.002
Emotional control	0 (Ref.)	−0.13 (−1.16, 0.91)	−0.96 (−1.97, 0.05)	−1.26 (−2.35, −0.17)^a^	0.004
MI	0 (Ref.)	0.07 (−0.97, 1.10)	−1.24 (−2.25, −0.23)^a^	−2.63 (−3.72, −1.54)^a^	<0.0001
Initiate	0 (Ref.)	−0.31 (−1.35, 0.72)	−1.56 (−2.57, −0.55)^a^	−2.84 (−3.92, −1.75)^a^	<0.0001
Working memory	0 (Ref.)	−0.11 (−1.13, 0.92)	−1.26 (−2.26, −0.26)^a^	−2.46 (−3.53, −1.39)^a^	<0.0001
Plan/organize	0 (Ref.)	−0.18 (−1.20, 0.84)	−1.30 (−2.30, −0.30)^a^	−2.45 (−3.53, −1.37)^a^	<0.0001
Organization of materials	0 (Ref.)	0.48 (−0.54, 1.51)	−0.87 (−1.87, 0.14)	−1.81 (−2.89, −0.72)^a^	<0.0001
Monitor	0 (Ref.)	0.23 (−0.79, 1.26)	−0.98 (−1.98, 0.03)	−1.95 (−3.02, −0.87)^a^	<0.0001
**Adjusted model**					
BRI	0 (Ref.)	0.18 (−0.86, 1.23)	−0.79 (−1.81, 0.24)	−1.07 (−2.17, 0.03)	0.006
Inhibit	0 (Ref.)	0.15 (−0.88, 1.17)	−0.61 (−1.61, 0.39)	−0.79 (−1.87, 0.29)	0.035
Shift	0 (Ref.)	0.26 (−0.80, 1.32)	−0.57 (−1.60, 0.47)	−1.05 (−2.17, 0.06)	0.008
Emotional control	0 (Ref.)	−0.06 (−1.12, 1.00)	−0.85 (−1.89, 0.19)	−1.09 (−2.20, 0.03)	0.012
MI	0 (Ref.)	0.32 (−0.73, 1.36)	−0.89 (−1.92, 0.13)	−2.13 (−3.24, −1.03)^a^	<0.0001
Initiate	0 (Ref.)	−0.17 (−1.22, 0.88)	−1.37 (−2.40, −0.34)^a^	−2.63 (−3.74, −1.53)^a^	<0.0001
Working memory	0 (Ref.)	0.19 (−0.85, 1.23)	−0.92 (−1.94, 0.10)	−2.04 (−3.13, −0.94)^a^	<0.0001
Plan/organize	0 (Ref.)	0.11 (−0.93, 1.15)	−0.93 (−1.94, 0.09)	−1.97 (−3.07, −0.87)^a^	<0.0001
Organization of materials	0 (Ref.)	0.53 (−0.52, 1.58)	−0.81 (−1.84, 0.22)	−1.55 (−2.66, −0.45)^a^	<0.0001
Monitor	0 (Ref.)	0.49 (−0.54, 1.52)	−0.62 (−1.63, 0.39)	−1.50 (−2.58, −0.41)^a^	0.0001

*BRI, behavioral regulation index; MI, metacognition index*.

*Crude model: no adjustment*.

*Adjusted model: adjusted for age, sex, only child, parental education, monthly family income, and screen time*.

a *Statistically significant association (p < 0.05)*.

In the sensitivity analysis, the associations between the number of ideal cardiovascular health metrics and executive function remained robust when using the Screening for overweight and obesity among school-age children and adolescents in 2018 (Supplementary Table S6).

### Individual Cardiovascular Health Metric and Executive Function

After additionally mutual adjustment for the remaining 6 health components, ideal diet was associated with 1.96 (95% *CI*: −2.67, −1.26) and 2.29 (95% *CI*: −2.99, −1.58) points reduction in BRI and MI, respectively, relative to the non-ideal levels (Table 3). Ideal MVPA was associated with 0.99 (95% *CI*: −1.67, −0.31) points lower in MI than the non-ideal MVPA.

**Table 3:** Association (95% *CI*) between individual cardiovascular health metric and executive function (*n* = 3,798).

**7 cardiovascular health metrics**	***β*** **(95% confidence interval)**	
	**BRI**	**MI**
**Ideal smoking**		
Crude model	−1.37 (−5.34, 2.60)	1.47 (−2.78, 5.71)
Adjusted model	−0.79 (−4.87, 3.28)	1.96 (−2.38, 6.29)
**Ideal BMI**		
Crude model	−0.18 (−0.97, 0.61)	−1.21 (−2.01, −0.41)^a^
Adjusted model	0.27 (−0.56, 1.11)	−0.83 (−1.66, 0.01)
**Ideal MVPA**		
Crude model	−0.72 (−1.38, −0.06)^a^	−1.20 (−1.87, −0.53)^a^
Adjusted model	−0.41 (−1.09, 0.27)	−0.99 (−1.67, −0.31)^a^
**Ideal diet**		
Crude model	−1.95 (−2.64, −1.26)^a^	−2.54 (−3.24, −1.85)^a^
Adjusted model	−1.96 (−2.67, −1.26)^a^	−2.29 (−2.99, −1.58)^a^
**Ideal total cholesterol**		
Crude model	−0.53 (−1.18, 0.12)	−0.61 (−1.26, −0.06)
Adjusted model	−0.43 (−1.09, 0.23)	−0.54 (−1.20, 0.12)
**Ideal blood pressure**		
Crude model	0.03 (−0.69, 0.75)	0.13 (−0.59, 0.86)
Adjusted model	0.08 (−0.66, 0.83)	0.39 (−0.35, 1.14)
**Ideal fasting blood glucose**		
Crude model	0.26 (−1.00, 1.53)	0.61 (−0.67, 1.90)
Adjusted model	0.08 (−1.21, 1.37)	0.34 (−0.95, 1.63)

*BRI, behavioral regulation index; MI, metacognition index*.

*Crude model: no adjustment*.

*Adjusted model: adjusted for age, sex, only child, parental education, monthly family income, screen time, and mutually adjusted for each other cardiovascular health metrics*.

a *Statistically significant association (p < 0.05)*.

### Effect Modification

We further evaluated potential modification effects of sex and SDQ behavioral problems group. No significant modification effects were observed for either sex or SDQ behavioral problems (Supplementary Table S7).

## Discussion

To our knowledge, this study is the first to apply the AHA construct of ideal cardiovascular health, generated from the 7 individual health factors and behaviors, to investigate its association with executive function in children. We observed a positive association between the number of ideal cardiovascular health metrics and executive function performance. These associations were independent of a number of potential confounding factors, such as demographic factors and behaviors. Additionally, these relationships appeared to be driven particularly by PA and diet, as the two variables were significantly associated with executive function after mutual adjustment for other cardiovascular health components.

The prevalence of children achieving all 7 ideal health metrics in this study was relatively low (4.0%), which is consistent with the findings of previous studies (3, 4). In an analysis of data from the National Health and Nutrition Examination Survey, no US children met all 7 criteria for ideal health metrics in 2015–2016 were reported (3). In a recent study of Chinese urban children, the prevalence of meeting all ideal components was only 0.5% (4). Poor diet (ideal diet: 31.6%) and physical inactivity (ideal PA: 38.8%) were the 2 worst cardiovascular health components in the present study, which were in line with an American study in adolescents (3) and another study in the Chinese urban children (4).

There is growing number of epidemiological studies to explore the associations between the individual or multiple components of the cardiovascular health and executive function in children. Poor diet, physical inactivity, obesity, and hypertension have each been shown to be associated with deficits in the executive function performance in children (9–11). The metabolic syndrome, characterized by a constellation of metabolic and anthropometric traits, such as abdominal obesity, hypertension, dyslipidemia, and hyperglycemia, has been reported to negatively impact the executive function in children (31). In addition, a greater number of behaviors, such as excess weight, physical inactivity, poor diet and sleep, and high screen time, has been documented to be associated with lower performance on mathematics, reading, and writing in children (32). However, studies exploring the relationship of combined cardiovascular health behaviors and metabolic factors with executive function are limited. Several previous studies applied the AHA-defined clusters of cardiovascular health metrics to explore its association with executive function, and all studies were conducted in adults or elderly populations (13–21). However, no literature on this topic in children has been performed. For example, several cross-sectional studies investigated the relationship of cardiovascular health status and cognitive function and found that higher cognitive function was associated with better cardiovascular health metrics among middle-aged and older adults (13, 17–20). Similarly, some other cohort studies from the United States revealed that a greater number of ideal cardiovascular metrics was associated with better cognitive function and low incidence of cognitive impairment in young adults and elderly participants (14–16). The findings of our study, in which a positive association was also observed between the number of ideal cardiovascular components and executive function performance in children, confirmed and extended prior research described above. Childhood is recognized as a vulnerable period for developing executive function (6). Our findings indicated that the achievement of AHA's ideal cardiovascular health was associated with the improving executive function in children. Future studies exploring the effects of ideal cardiovascular health on executive function in children are needed.

Physical activity and diet were positively associated with executive function performance in this study. Mounting evidence from prospective observational studies and randomized clinical trials have demonstrated the beneficial effects of PA on cognitive function in children and adolescents (33). The mechanisms linking the two are still unclear, one plausible pathway is through the stimulation of some factors involved in brain plasticity by PA (34). Exercise increases the formation of new neurons and concentrations of brain-derived neurotrophic factor, improves cerebral blood flow and oxygen availability in the brain, as well as enhances activity-dependent synaptic plasticity (34). This set of physiological changes might lead to improve performance on cognition. Similarly, the association between diet and executive function has been reported in literatures (35). The healthy diet characterized by high consumption of fruits, vegetable, fish, milk, and beans, as well as low intake of fried food and SSB was assessed in this study. Several possible mechanisms underlying the beneficial effects of diet on cognitive function have been proposed. For instance, antioxidants and soluble fiber rich in fruit and vegetables might contribute to ameliorate oxidative stress (i.e., caused by free radicals) and proliferate health-promoting bacteria separately, thereby protecting neural cells from damage (36). In addition, soy foods are the major source of isoflavones, which have been reported to have neuroprotective effects owing to their antioxidant and anti-inflammatory effects, as well as their interactions with estrogen receptors localized throughout the brain (37).

The present study has some limitations. First, the cross-sectional study design prevented us from inferring the temporality of the ideal cardiovascular health metrics and executive function. Second, although we were able to adjust for several factors known to be important for the executive function of children, it is possible that the observed associations were biased by unmeasured confounding factors, such as pubertal status and parental cognitive status. Third, only the parental reports of behavioral executive function, a tool for ecological assessment, was used in the study, which could result in information bias. The computerized testing, a standard and systematic method for cognitive assessment, could be used with questionnaire together to complement each other's advantages and comprehensively assess executive function (38). Fourth, we modified the AHA definitions of diet component based on the data available in this study, which potentially altered the comparability of results to similar analyses in other studies.

## Conclusion

In conclusion, we found that the number of ideal cardiovascular health was positively associated with performance of executive function in children. Public health practitioners and parents should encourage children to maintain better cardiovascular health profiles to improve executive function.

## Data Availability Statement

The raw data supporting the conclusions of this article will be made available by the authors, without undue reservation.

## Ethics Statement

The studies involving human participants were reviewed and approved by Ethical Review Committee for Biomedical Research, Sun Yat-sen University. Written informed consent to participate in this study was provided by the participants' legal guardian/next of kin.

## Author Contributions

YC, LC, and ZG designed this study. ZG, YL, LL, and XZ contributed to the data collection. ZG analyzed the data and drafted the manuscript. YC, LC, YL, LL, and XZ critically reviewed the manuscript. All authors read and approved the final manuscript.

## Funding

The work was supported by the National Natural Science Foundation of China (Grant numbers 81673193).

## Conflict of Interest

The authors declare that the research was conducted in the absence of any commercial or financial relationships that could be construed as a potential conflict of interest.

## Publisher's Note

All claims expressed in this article are solely those of the authors and do not necessarily represent those of their affiliated organizations, or those of the publisher, the editors and the reviewers. Any product that may be evaluated in this article, or claim that may be made by its manufacturer, is not guaranteed or endorsed by the publisher.
