# Supplementary material for: Association Between Ideal Cardiovascular Health and Executive Function in Chinese Primary School Children
Source: Front Public Health. 2022 Jan 12;9:736424. doi: 10.3389/fpubh.2021.736424 (PMC8790157; doi:10.3389/fpubh.2021.736424)
Supplement: Supplementary file 1 [file Table_1.DOCX]

**Supplemental Materials**

**Table of Contents**

**Table S1.** Definitions of poor, intermediate, and ideal Levels of 7 cardiovascular health metrics in children (adapted from the American Heart Association 2020 Impact Goals)

**Table S2.** Prevalence of cardiovascular health metrics (n, %)

**Table S3.** Prevalence of meeting the goal for individual component of healthy diet score in children (n, %)^a^

**Table S4.** Associations (95% confidence interval) between ideal cardiovascular health metrics and executive function, stratified by potential modifiers (n=3,798)

**Table S1.** Definitions of poor, intermediate, and ideal Levels of 7 cardiovascular health metrics in children (adapted from the American Heart Association 2020 Impact Goals)

| Metrics | Poor Health | Intermediate Health | Ideal Health |
| --- | --- | --- | --- |
| Current smoking | Tried prior 30 days | − | Never tried; never smoked whole cigarette |
| Body mass index | ≥ 95th Percentile | 85th − 95th Percentile | < 85th Percentile |
| Physical activity | None | 1-59 min of moderate or vigorous activity every day | ≥ 60 min of moderate- or vigorous- intensity activity every day |
| Healthy diet score^a^ | 0 − 1 components | 2 − 3 components | 4 − 5 components |
| Total cholesterol | ≥ 5.2 mmol/L | 4.4 − 5.1 mmol/L | < 4.4 mmol/L |
| Blood pressure | ≥ 95th Percentile | 90th − 95th Percentile | < 90th Percentile |
| Fasting plasma glucose | ≥ 7.1 mmol/L | 5.6 − 7.0 mmol/L | < 5.6 mmol/L |

^a^ The healthy diet score included 5 dietary criteria goals: daily both fruits and vegetable consumption ≥ 1 times, fish or fish products ≥ 2 times/week, daily milk, beans or dairy-or bean-products consumption ≥ 1 times, fried food ≤ 2 times/week, and sugar-sweetened beverages ≤ 2 times/week.

Reference:

1. Lloyd-Jones DM, Hong Y, Labarthe D, Mozaffarian D, Appel LJ, Van Horn L, Greenlund K, Daniels S, Nichol G, Tomaselli GF, Arnett DK, Fonarow GC, Ho PM, Lauer MS, Masoudi FA, Robertson RM, Roger V, Schwamm LH, Sorlie P, Yancy CW, Rosamond WD. Defining and setting national goals for cardiovascular health promotion and disease reduction: the American Heart Association's strategic Impact Goal through 2020 and beyond. *Circulation* 2010;121:586-613.

**Table S2.** Prevalence of cardiovascular health metrics (n, %)

|  | All (n=3,798) | Boys (n=1,981) | Girls (n=1,817) |
| --- | --- | --- | --- |
| Each individual health metric |  |  |  |
| ***Health behavior*** |  |  |  |
| Smoking status |  |  |  |
| Poor (Tries prior to 30 d) | 25 (0.7) | 16 (0.8) | 9 (0.5) |
| Ideal (Never tried) | 3,773 (99.3) | 1965 (99.2) | 1808 (99.5) |
| Body mass index^a^ |  |  |  |
| Poor (> 95th percentile) | 362 (9.5) | 247 (12.5) | 115 (6.3) |
| Intermediate (85 − 95th percentile) | 438 (11.5) | 301 (15.2) | 137 (7.5) |
| Ideal (< 85th percentile) | 2,998 (78.9) | 1,433 (72.3) | 1565 (86.1) |
| MVPA^a^ |  |  |  |
| Poor (0 min/d) | 146 (3.8) | 70 (3.5) | 76 (4.2) |
| Intermediate (0 − 59 min/d) | 2,178 (57.3) | 1075 (54.3) | 1103 (60.7) |
| Ideal (≥ 60 min/d) | 1,474 (38.8) | 836 (42.2) | 638 (35.1) |
| Healthy diet score^a,b^ |  |  |  |
| Poor (0 − 1 components) | 107 (2.8) | 65 (3.3) | 42 (2.3) |
| Intermediate (2 − 3 components) | 2,491 (65.6) | 1354 (68.3) | 1,137 (62.6) |
| Ideal (4 − 5 components) | 1,200 (31.6) | 562 (28.4) | 638 (35.1) |
| ***Health factors*** |  |  |  |
| Total cholesterol^a^ |  |  |  |
| Poor (≥ 5.2 mmol/L) | 484 (12.7) | 224 (11.3) | 260 (14.3) |
| Intermediate (4.4 − 5.2 mmol/L) | 1,286 (33.9) | 682 (34.4) | 604 (33.2) |
| Ideal (< 4.4 mmol/L) | 2,028 (53.4) | 1075 (54.3) | 953 (52.4) |
| Blood pressure |  |  |  |
| Poor (> 95th percentile) | 671 (17.8) | 360 (18.3) | 311 (17.2) |
| Intermediate (90 − 95th percentile) | 443 (11.7) | 232 (11.8) | 211 (11.7) |
| Ideal (< 90th percentile ) | 2,663 (70.5) | 1376 (69.9) | 1287 (71.1) |
| Fasting blood glucose^a^ |  |  |  |
| Poor (≥ 7.1 mmol/L) | 3 (0.1) | 1 (0.1) | 2 (0.1) |
| Intermediate (5.6 − 7.0 mmol/L) | 281 (7.4) | 176 (8.8) | 105 (5.8) |
| Ideal (< 5.6 mmol/L) | 3,514 (92.5) | 1804 (91.1) | 1710 (94.1) |
| ***Number of ideal health components***^a^ |  |  |  |
| 1 | 7 (0.2) | 5 (0.3) | 2 (0.1) |
| 2 | 112 (2.9) | 77 (3.9) | 35 (1.9) |
| 3 | 446 (11.7) | 263 (13.3) | 183 (10.1) |
| 4 | 1,099 (28.9) | 573 (28.9) | 526 (28.9) |
| 5 | 1,271 (33.5) | 644 (32.5) | 627 (34.5) |
| 6 | 711 (18.7) | 342 (17.3) | 369 (20.3) |
| 7 | 152 (4.0) | 77 (3.9) | 75 (4.1) |
| ***Number of ideal health behaviors***^a^ |  |  |  |
| 0 | 3 (0.1) | 3 (0.2) | 0 |
| 1 | 343 (9.0) | 228 (11.5) | 115 (6.3) |
| 2 | 1,674 (44.1) | 879 (44.4) | 795 (43.8) |
| 3 | 1,358 (35.8) | 674 (34.0) | 684 (37.6) |
| 4 | 420 (11.1) | 197 (9.9) | 223 (12.3) |
| ***Number of ideal health factors***^a^ |  |  |  |
| 0 | 50 (1.3) | 37 (1.9) | 13 (0.7) |
| 1 | 628 (16.5) | 334 (16.9) | 294 (16.2) |
| 2 | 1,783 (46.9) | 909 (45.9) | 874 (48.1) |
| 3 | 1,337 (35.2) | 701 (35.4) | 636 (35.0) |

Abbreviations: MVPA, moderate-to-vigorous-intensity physical activity.

^a^ Statistically significant difference between boys and girls (*p* <0.05).

^b^ The healthy diet score included 5 dietary criteria goals: daily both fruits and vegetable consumption ≥ 1 times, fish or fish products ≥ 2 times/week, daily milk, beans or dairy-or bean-products consumption ≥ 1 times, fried food ≤ 2 times/week, and sugar-sweetened beverages ≤ 2 times/week.

**Table S3.** Prevalence of meeting the goal for individual component of healthy diet score in children (n, %)^a^

| Component | All | Boys | Girls |
| --- | --- | --- | --- |
| Fruits and vegetables^b^ | 1,618 (42.6) | 792 (40.0) | 826 (45.5) |
| Fish or fish products | 2,842 (74.8) | 1,462 (73.8) | 1,380 (75.9) |
| Bean- or dairy-products^b^ | 123 (3.2) | 71 (3.6) | 52 (2.9) |
| Fried food | 3,681 (96.9) | 1,923 (97.1) | 1,758 (96.8) |
| Sugar-sweetened beverages^b^ | 3,409 (89.8) | 1,736 (87.6) | 1,673 (92.1) |

^a^ The healthy diet score included 5 dietary criteria goals: daily both fruits and vegetable consumption ≥ 1 times, fish or fish products ≥ 2 times/week, daily milk, beans or dairy-or bean-products consumption ≥ 1 times, fried food ≤ 2 times/week, and sugar-sweetened beverages ≤ 2 times/week.

^b^ Statistically significant difference between boys and girls (*p* <0.05).

**Table S4.** Associations (95% confidence interval) between ideal cardiovascular health metrics and executive function, stratified by potential modifiers (n=3,798)

| Group |  | *β* (95% confidence interval)^a^ | |
| --- | --- | --- | --- |
|  |  | BRI | MI |
| Sex | Boys |  |  |
|  | 1 to 3 points | 0 (Ref.) | 0 (Ref.) |
|  | 4 points | 0.03 (-1.40, 1.46) | 0.32 (-1.10, 1.73) |
|  | 5 points | -0.71 (-2.11, 0.68) | -0.97 (-2.35, 0.41) |
|  | 6 to 7 points | -0.54 (-2.08, 1.01) | -1.87 (-3.40, -0.35) |
|  | Girls |  |  |
|  | 1 to 3 points | 0 (Ref.) | 0 (Ref.) |
|  | 4 points | 0.35 (-1.22, 1.91) | 0.34 (-1.24, 1.93) |
|  | 5 points | -0.88 (-2.41, 0.66) | -0.83 (-2.38, 0.72) |
|  | 6 to 7 points | -1.49 (-3.10, 0.13) | -2.30 (-3.93, -0.67) |
|  | *P*_-interaction_ | 0.50 | 0.92 |
| SDQ behavioral  problems |  |  |  |
|  | Borderline/abnormal |  |  |
|  | 1 to 3 points | 0 (Ref.) | 0 (Ref.) |
|  | 4 points | 0.40 (-4.35, 5.15) | 0.23 (-3.69, 4.15) |
|  | 5 points | -3.49 (-8.06, 1.09) | -1.40 (-5.15, 2.34) |
|  | 6 to 7 points | -1.72 (-7.11, 3.67) | -0.73 (-5.19, 3.72) |
|  | Normal |  |  |
|  | 1 to 3 points | 0 (Ref.) | 0 (Ref.) |
|  | 4 points | -0.02 (-1.03, 0.98) | 0.17 (-0.89, 1.24) |
|  | 5 points | -0.89 (-1.87, 0.09) | -1.12 (-2.16, -0.08) |
|  | 6 to 7 points | -0.98 (-2.03, 0.07) | -2.26 (-3.37, -1.14) |
|  | *P*_-interaction_ | 0.14 | 0.88 |

Abbreviations: BRI, behavioral regulation index; MI, metacognition index; SDQ, Strengths and Difficulties Questionnaire.

All analyses were adjusted for age, sex, only child, parental education, monthly family income, and screen time.
